# Significance of Polymorphism and Expression of miR-146a and *NFkB1* Genetic Variants in Patients with Rheumatoid Arthritis

**DOI:** 10.1007/s00005-016-0443-5

**Published:** 2017-01-12

**Authors:** Katarzyna Bogunia-Kubik, Barbara Wysoczańska, Dagmara Piątek, Milena Iwaszko, Marzena Ciechomska, Jerzy Świerkot

**Affiliations:** 1 Laboratory of Clinical Immunogenetics and Pharmacogenetics, Hirszfeld Institute of Immunology and Experimental Therapy, Polish Academy of Sciences, Wroclaw, Poland; 2 Department of Internal, Occupational Diseases, Hypertension and Clinical Oncology, Wroclaw Medical University, Wroclaw, Poland; 3 Department of Rheumatology and Internal Medicine, Wroclaw Medical University, Borowska 213, 50-556 Wroclaw, Poland; 4 National Institute of Geriatrics Rheumatology and Rehabilitation, Warsaw, Poland

**Keywords:** Rheumatoid arthritis, *miRNA*-*146a*-*3p* polymorphism, miRNA-146a-5p serum level, *NFkB1* polymorphism, Disease susceptibility, Response to treatment

## Abstract

MicroRNA-146a (miR-146a) has been shown to play an important role in the regulation of inflammatory innate immune responses, and found to be differentially expressed in rheumatoid arthritis (RA). Through NF-κB pathway, this molecule is able to stimulate the release of pro-inflammatory cytokines such as TNF-α, IL-1β, and IL-17. It has been also suggested that single-nucleotide polymorphisms (SNPs) in miRNA sequences may alter miRNA expression and that miR-146a rs2910164 SNP may contribute to RA development. These observations prompted us to analyze the potential associations between the *miR*-*146a*-*3p* (rs2910164, G > C) and *NFkB1* (rs28362491, ins/del ATTG) polymorphisms and miR-146a-5p expression in patients’ sera in relation to clinical outcome of the treatment as well as predisposition to RA. Genotyping was performed in 111 patients and 130 healthy individuals while 16 controls and 13 RA patients (before and after three months of therapy with TNF-α inhibitors (TNFi)) were studied for the circulating miR-146a-5p serum expression level. Patients carrying the *NFkB1 ins/ins* genotype were characterized by worse response to TNFi treatment (*p* = 0.023). In patients, before TNFi therapy, expression levels of miR-146a-5p were less (0.422 ± 0.171) as compared to those detected after three months of treatment (1.809 ± 0.658, *p* = 0.033) and observed for healthy controls (5.302 ± 2.112, *p* = 0.048). Moreover, patients with higher circulating miR-146a-5p levels after three months of TNFi administration were more frequently carrying the rs2910164-*C* allele (*p* = 0.032). These results support the hypothesis that miR-146a might be involved in pathogenesis of RA and imply that *miR*-*146a*-*3p* polymorphism may be associated with miR-146a-5p levels in serum after anti-TNF-α treatment.

## Introduction

Rheumatoid arthritis (RA) is a systemic, inflammatory autoimmune disease primarily characterized by chronic synovitis and progressive joint destruction. The exact cause of RA remains unknown, however, it has been shown that both genetic and environmental factors play a role in the disease development (McInnes and Schett 2011). Anti-tumor necrosis factor (anti-TNF) biologic agents represent a novel approach in RA management that significantly improved the prognosis of RA patients. Although, substantial proportion of patients do not respond to the therapy with TNF inhibitors. The reasons of anti-TNF therapy failure have not been established to date. The search of biomarkers of anti-TNF agents’ efficacy is of importance to optimize patient benefit and reduce cost of treatment.

MicroRNAs (miRNAs, miRs) and nuclear factor kappa-light-chain-enhancer of activated B cells (NF-κB) are well-known immune response and inflammation regulators.

MiRNAs are a family of single-stranded, non-coding endogenous regulatory molecules, cleaved from double stranded precursors, typically composed of 21–23 nucleotides. They are involved in the regulation of gene expression mainly at posttranscriptional level (Bartel 2009). It is estimated that the genes encoding the miRNA constitute 1–5% of the genes in humans and animals. A total of 2588 mature miRNAs were identified in humans (Eulalio and Mano 2015). More than 30% of protein encoding genes in human cells are regulated by miRNA (Krol et al. 2010). A single miRNA molecule can simultaneously control the expression of hundreds of target genes.

MiRNAs are known to be the target for NF-κB transcriptional control and at the same time are involved in the modulation of NF-κB signaling (Boldin and Baltimore 2012; Ghosh and Hayden 2008).

NF-κB regulates numerous pro-inflammatory cytokines, chemokines, and adhesion molecules involved in the activation and recruitment of inflammatory modulating cells (Ghosh and Hayden 2008). Patients with RA present constitutively high serum levels of pro-inflammatory cytokines, including TNF-α, IL-1β, IL-6 or IL-17, which are known to be NF-κB target genes, suggesting activation of this signaling pathway in the course of disease.

Our former studies showed that genetic variability within genes coding for pro-inflammatory cytokines (Bogunia-Kubik et al. 2015; Świerkot et al. 2015) may play a role in RA development and response to treatment with TNF-α inhibitors (TNFi). It has been also documented that acting through NF-κB pathway, some miRNAs such as miR-146a and miR-155 may stimulate the release of pro-inflammatory cytokines. As the inflammatory mediators, these cytokines can induce lymphocytes, resident synovial cells, and other inflammatory cells to produce miRNAs that are related to disease activity of rheumatic disorders as shown for RA patients (Chen et al. 2015).

The miR-146 acts as a negative regulator of the TLR/NF-κB signaling pathway. It was reported that the NF-κB activation may induce expression of the miR-146. On the other hand, miR-146 affects the expression of TNF receptor-associated factor 6 (TRAF6) and IL-1 receptor-associated kinase 1 (IRAK1), that constitute key adapter molecules involved in the Toll-like receptor (TLR)/NF-κB pathway. MiR-146-dependent downregulation of the TRAF6 and IRAK1 may result in inhibition of the TLR/NF-κB signaling axis (Taganov et al. 2006). The G > C substitution (rs2910164) at position +60 relative to the first nucleotide of the precursor *mir*-*146a* is a potentially functional single-nucleotide polymorphism (SNP) within the *pre*-*mir*-*146a* (5q33) gene that also occurs in the 3p strand in mature miRNA. This SNP affects the amount of pre- and mature miRNA-146 through disruption of miRNA-146 processing (Jazdzewski et al. 2008). A direct functional effect of the rs2910164 polymorphism on the miRNA-146a capacity to inhibit its target genes (TRAF6 and IRAK1) has been also revealed (Jazdzewski et al. 2008). Taking into account that TRAF6 and IRAK1 have been implicated in RA pathogenesis, this polymorphism may contribute to RA development (Chatzikyriakidou et al. 2010).

There is a common insertion/deletion (–94 ins/del ATTG) polymorphism located within the *NFkB1* promoter (rs28362491) exerting functional effects on the transcription of the gene (Karban et al. 2004). This gene is located on chromosome 4q24 and encodes subunits p105 and p50 kD of NF-κB. The p50 homodimer represses transcription of pro-inflammatory cytokines and stimulates transcription of anti-inflammatory cytokines (Cartwright et al. 2016).

The aim of the present study was to analyze potential associations between *NFkB1* (rs28362491, ins/del ATTG) and *miR*-*146a*-*3p* (rs2910164, G > C) polymorphisms and miR-146a-5p expression in patients’ sera in relation to clinical outcome of the treatment as well as predisposition to RA.

## Materials and Methods

### Genotyping Studies

DNA was isolated from peripheral blood of 111 RA patients hospitalized at the Rheumatology Clinic of the Medical University in Wroclaw and 130 healthy individuals that served as a control group for disease association studies. Patients and controls were genotyped for the *miR*-*146a*-*3p* (rs2910164, G > C) alleles using a polymerase chain reaction (PCR) amplification employing the LightSNiP assay (TIB MOLBIOL, Berlin, Germany). Capillary electrophoresis or PCR followed by restriction fragment length polymorphism (PCR–RFLP with *Pfl*Ml digestion) were employed to study the *NFkB1* (rs28362491, ins/del ATTG) alleles, as previously described by Zhou et al. (2009) and Koc et al. (2014), respectively. Separation of PCR products was performed in a 50 cm 8 capillary array containing POP-7 polymer on 3500 Genetic Analyzer (Applied Biosystem, USA) and analyzed by GeneMapper Software v 4.2 (Applied Biosystem, USA). Both techniques gave the same results with 100% concordance in duplicate samples.

The study was approved by the Wroclaw Medical University Ethics Committee and written informed consent was obtained from all participants.

### Analysis of Circulating miR-146a-5p Expression

For analysis of the miR-146a-5p expression, RNA was isolated from sera of 13 patients (before and three months after anti-TNF-α treatment) and 16 healthy controls with the use of Nucleospin^®^ miRNA Plasma (MACHEREY–NAGEL GmbH&Co.KG). Reverse transcription was conducted using TaqMan^®^ MicroRNA Reverse Trascription Kit Cat. # 4,366,596 (Applied Biosystems, Life Technologies), in accordance with the manufacture’s protocol. The reaction was carried out in a SimpliAmpTM Thermal Cycler (Applied Biosystems, Life Technologies) at 16 °C for 30 min, 42 °C for 30 min, and 85 °C for 5 min. The product of reverse transcription was stored at –20 °C until further use.

Expression of miR-146a-5p was analyzed by Real Time PCR. The reaction was performed on a ViiaTM 7 Real Time PCR System (Applied Biosystems) using the TaqMan microRNA Assay quantitate miRNAs: hsa-miR-146a-5p Cat. # 4,427,975 primers for human miR-146a-5p and U6 together with TaqMan Universal PCR Master Mix II, no UNG Cat. # 4,440,040 (Applied Biosystems). MiR-146a-5p expression was normalized to U6, which was endogenous small nuclear RNA control (TaqMan MicroRNA Assays, Applied Biosystems). All reactions were carried out in duplicates. The results were analyzed using the (ΔΔCt) calculations. The data are presented as mean ± SEM.

### Statistical Analysis

All genotypes were tested for deviations from Hardy–Weinberg equilibrium (HWE) using the *χ* ^2^ test. Fisher’s exact test was used to compare the allele and genotype frequencies between patients and controls.

The differences in miR-146a-5p serum expression levels between the groups were tested by non-parametric two-tailed *T* test or Wilcoxon matched-pairs rank test. A *p* value of less than 0.05 was considered statistically significant.

## Results

### Distribution of the miR-146a and *NFkB1* Genotypes in Patients and Controls

We found no evidence that genotype frequencies of the two polymorphisms examined were different from those expected from HWE both in controls and cases. There was no linkage disequilibrium between the studied polymorphisms. Genotype distributions of both SNPs were similar between patients and controls (Table 1). Thus, neither the rs2910164 *miR*-*146a*-*3p* nor the rs28362491 *NFkB1* polymorphism was found to be associated with predisposition to RA. Furthermore, no significant relationship was detected for any of parameters such as: anti-cyclic citrullinated peptide antibodies, rheumatoid factor, C-reactive protein and disease activity score (DAS28) (individual data not shown).

**Table 1:** Distribution of rs2910164 *miR*-*146a*-*3p* and rs28362491 *NFkB1* genotypes in RA patients and controls

Gene	Polymorphism	Genotype	RA patients	Controls
*miR*-*146a*-*3p*	rs2910164	GG	72 (65%)	88 (68%)
		GC	32 (29%)	36 (28%)
		CC	7 (6%)	6 (4%)
*NFkB1*	rs28362491	*ins/ins*	36 (33%)	43 (34%)
		*ins/del*	55 (50%)	69 (55%)
		*del/del*	19 (17%)	14 (11%)

### Response to TNFi Treatment

Clinical response was evaluated according to the European League Against Rheumatism criteria at the third month after initiation of the TNFi therapy (Fransen and van Riel 2005).

**Fig. 1:**
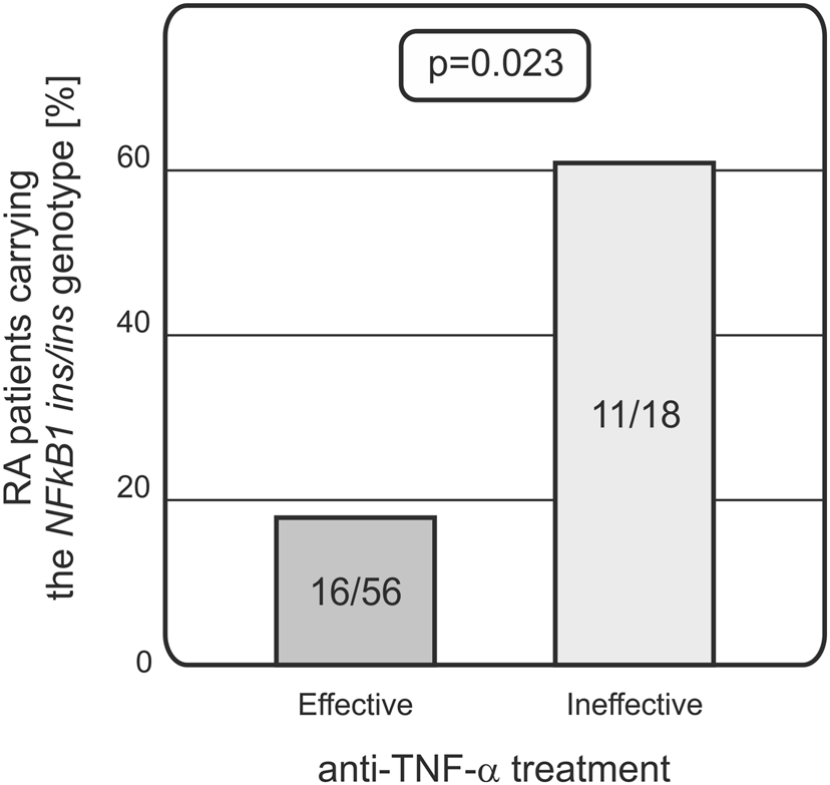
Relationship between the *NFkB1* polymorphism and response to treatment in RA patients

The *NFkB1* ins/del polymorphism was found to be associated with response to the biological treatment. Patients homozygous for the *ins* allele appeared to be worse responders as compared to the *del* allele carriers. The *ins/ins* genotype was detected in 11 out of 18 (61%) of patients with unsuccessful outcome of the treatment and only in 16 out of 56 (18%) of those for whom the therapy was successful (*p* = 0.023; Fig. 1).

**Fig. 2:**
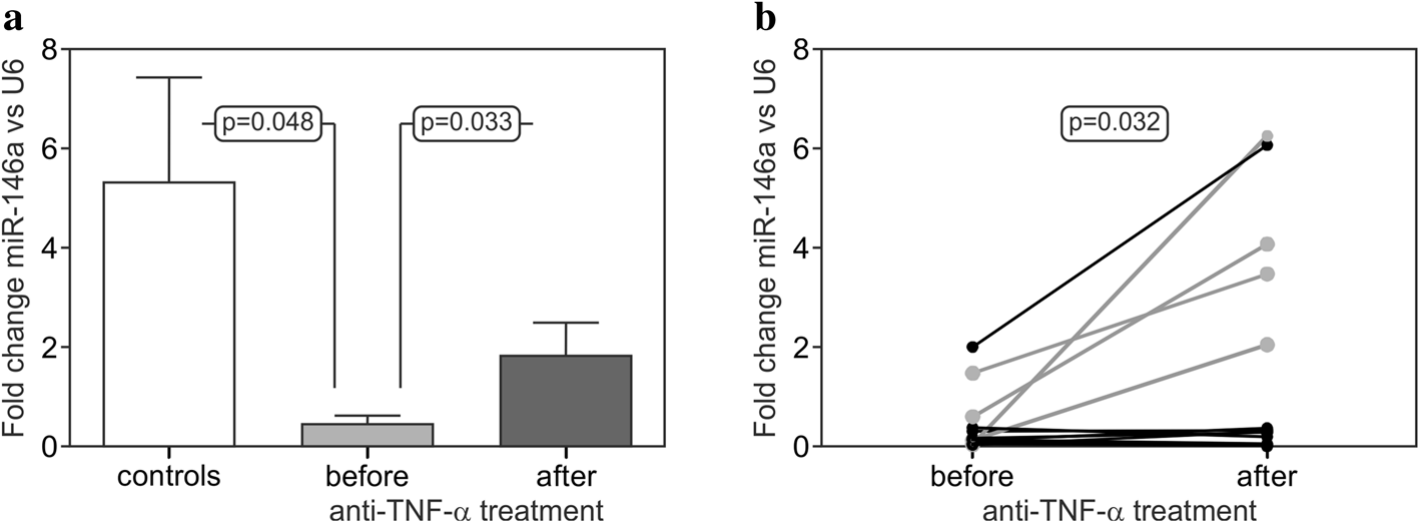
Serum expression levels of miR-146a-5p in RA patients (before and after 3 months of TNFi treatment) and controls (**a**) and changes in miR-146-5p expression profile in RA patients with respect to the presence of the *C* polymorphic variant (*marked in gray*) of the *miR*-*146a*-*3p* (rs2910164) SNP (**b**)

Moreover, some significant differences were observed with respect to the expression levels of circulating miR-146a-5p. Patients after three months of TNFi administration had higher miR-146a-5p levels in serum than those before the treatment (4.3-fold increase; 1.809 ± 0.658 vs 0.422 ± 0.171, *p* = 0.033; Fig. 2a) and they were more frequently carrying the *miR*-*146a C* allele (the gray dots on the graph; Fig. 2b). Four out of five patients (80%) carrying the *C* variant presented with increased serum levels after TNFi treatment as compared to only one out of eight (12.5%) of the *GG* homozygous patients (*p* = 0.032; Fig. 2b). Expression was higher in controls as compared to the patients, especially those before TNFi treatment (2.9-fold; 5.302 ± 2.112 vs 1.809 ± 0.658, *p* = 0.048; Fig. 2a). The *NFkB1 ins*/*del* polymorphism was not found to affect the serum expression level of circulating miR-146a.

## Discussion

The results of the present study suggest that miR-146a might play an important role in the pathogenesis of RA. Serum expression levels of miR-146a-5p were significantly reduced in patients as compared to healthy individuals. Moreover, increase of miR-146a-5p expression levels was observed in patients after three months of anti-TNF-α therapy. This observation indicates that administration of anti-TNF-α drugs gradually increases the miR-146-5p level in patients’ sera, potentially up to the level observed for healthy controls. Our data also imply that *miR*-*146a*-*3p* rs2910164 polymorphism may be associated with miR-146a-5p levels in serum after TNFi treatment, with higher levels observed for the *C* allele carriers. However, this genetic variant did not influence the predisposition to RA or efficacy of anti-TNF-α therapy.

In line with the results of our present study, the reduced miR-146a serum expression levels have been previously reported by Filková et al. (2014) in patients with early RA as compared to healthy controls. Also, significantly reduced serum levels of miRNA-146a were observed in patients with established RA (Wang et al. 2012). Moreover, changes of miR-146a levels in sera of RA patients before and after anti-TNF treatment have been investigated in the study by Castro-Villegas et al. (2015). Consistent with our results, serum expression levels of the miR-146a were significantly upregulated in patients following anti-TNF therapy (Castro-Villegas et al. 2015). On the other hand, elevated expression of the miR-146a was observed in synovial tissues, synovial fluid monocytes, peripheral blood-derived mononuclear cells, and serum from RA patients (Murata et al. 2010; Pauley et al. 2008).

The previous studies documented that the *miR*-*146a* SNP may be associated with predisposition to metabolic syndrome development (Mehanna et al. 2015) or carcinogenesis (Jazdzewski et al. 2008; Wojcicka et al. 2014). As for the *miR*-*146a* SNP, some associations with pathogenesis of rheumatic diseases have been described especially for patients with ankylosing spondylitis in a Han Chinese population (Xu et al. 2015). However, this SNP has not been reported to be associated with RA in either Asian or Caucasian populations (Chatzikyriakidou et al. 2010; El-Shal et al. 2013; Zhou et al. 2015; the present study). Although, in a study of Zhou et al. (2015), a significant association of the *GG* genotype with RA in females was observed, while the *CC* homozygosity seemed to be correlated with the DAS28 score.

With respect to the *NFkB1* (rs28362491, ins/del ATTG) polymorphism, the previous studies documented that it may affect the susceptibility to various diseases, e.g., cancer (Bu et al. 2007; Cartwright et al. 2016), autoimmune disorders including ulcerative colitis (Karban et al. 2004) or cardiovascular disease in patients with RA (López-Mejías et al. 2012).

Our genotyping results seem to be in agreement with those previously published. The frequency of the *del* allele was formerly reported to vary 32–54% between various ethnic populations, compared with 42 and 39% for patients and controls of the present study (Amador et al. 2016; Koc et al. 2014). However, we did not find any association with predisposition to RA. Nevertheless, we did observe some relationships with the treatment outcome.

The *ins/ins* homozygosity was found to be associated with worse response to therapy with TNFi. Interestingly, a comparison of the differences in miR-146a serum levels before and three months after TNFi treatment showed that patients lacking this *ins/ins* homozygous genotype had over three times higher mean difference between serum levels at these two time points (1.748 vs 0.558 pg/mL, *p* = 0.093) as compared to those carrying *ins/ins* homozygosity that were characterized by similar circulating miR-146a levels.

Results from the in vitro functional study (Karban et al. 2004) suggest that the presence of the deletion may be associated with diminished expression of the gene, leading to reduced p50/p105 NF-κB protein production. Since p50 has been shown to repress the production of pro-inflammatory cytokines, including TNF-α (Pereira and Oakley 2008), beneficial role of *ins* genotype in context of anti-TNF treatment outcome may be expected.

Indeed, it was observed that the presence of the *del* allele enhances production of pro-inflammatory cytokines such as, for example, IL-6 (44.23 vs 14.80 pg/mL, for *del/del* vs *ins/ins* genotypes; Koc et al. 2014) or TNF-α (91.32 vs 66.10 vs 40.73 pg/mL, for *del/del*, *del/ins*, *ins/ins* genotype carriers, respectively; our unpublished results).

However, in the present study, the *ins/ins* genotype correlated with worse response to anti-TNF therapy. Nonetheless, it should be noted that in vitro studies involving a restricted set of biological factors may not reflect interactions occurring in vivo in a disease environment. Furthermore, the *ins* variant has been previously documented as a risk factor of other autoimmune-related diseases such as psoriasis (Li et al. 2008) or Behcet’s disease (Yenmis et al. 2015). Moreover, recently performed meta-analysis concerning a role of the polymorphism in autoimmune disorders revealed the favorable effect of the *del* allele (Zou et al. 2011).

In summary, these preliminary results support the hypothesis that miR-146a might be involved in pathogenesis of RA as differences in serum levels were observed during therapy with TNF-α inhibitors. The results of the present study also suggest that the miR-146a polymorphism may be associated with miRNA levels in serum after anti-TNF-α treatment while the *NFkB1* polymorphism may affect the efficacy of the therapy. Obviously, these observations should be confirmed in a more extensive study. More research is needed to delineate the mechanism of NF-κB and miR-146a action underlying inflammatory response in RA.
